# Prognostic Biomarker DDOST and Its Correlation With Immune Infiltrates in Hepatocellular Carcinoma

**DOI:** 10.3389/fgene.2021.819520

**Published:** 2022-01-31

**Authors:** Changyu Zhu, Hua Xiao, Xiaolei Jiang, Rongsheng Tong, Jianmei Guan

**Affiliations:** ^1^ Department of Pharmacy, Sichuan Academy of Medical Science and Sichuan Provincial People’s Hospital, School of Medicine, University of Electronic Science and Technology of China, Chengdu, China; ^2^ Personalized Drug Therapy Key Laboratory of Sichuan Province, Sichuan Academy of Medical Sciences and Sichuan Provincial People’s Hospital, Chengdu, China; ^3^ Department of Pharmacy, Sichuan Academy of Medical Sciences and Sichuan Provincial People’s Hospital, Chengdu, China; ^4^ Department of Pharmacy, Gansu Provincial Hospital of TCM, Lanzhou, China; ^5^ Central Sterile Supply Department, Sichuan Academy of Medical Sciences and Sichuan Provincial People’s Hospital, Chengdu, China

**Keywords:** DDOST, HCC, tumor-infiltration, prognosis, T helper cells

## Abstract

**Background:** Dolichyl-diphosphooligosaccharide–protein glycosyltransferase non-catalytic subunit (DDOST) is an important enzyme in the process of high-mannose oligosaccharide transferring in cells. Increasing DDOST expression is associated with impairing liver function and the increase of hepatic fibrosis degrees, hence exacerbating the liver injury. However, the relation between DDOST and hepatocellular carcinoma (HCC) has not been revealed yet.

**Method:** In this study, we evaluated the prognostic value of DDOST in HCC based on data from The Cancer Genome Atlas (TCGA) database. The relationship between DDOST expression and clinical-pathologic features was evaluated by logistic regression, the Wilcoxon signed-rank test, and Kruskal–Wallis test. Prognosis-related factors of HCC including DDOST were evaluated by univariate and multivariate Cox regression and the Kaplan–Meier method. DDOST-related key pathways were identified by gene set enrichment analysis (GSEA). The correlations between DDOST and cancer immune infiltrates were investigated by the single-sample gene set enrichment analysis (ssGSEA) of TCGA data.

**Results:** High DDOST expression was associated with poorer overall survival and disease-specific survival of HCC patients. GSEA suggested that DDOST is closely correlated with cell cycle and immune response *via* the PPAR signaling pathway. ssGSEA indicated that DDOST expression was positively correlated with the infiltrating levels of Th2 cells and negatively correlated with the infiltration levels of cytotoxic cells.

**Conclusion:** All those findings indicated that DDOST was correlated with prognosis and immune infiltration in HCC.

## Introduction

As the principal histologic type of liver cancer, hepatocellular carcinoma (HCC) ranks third among the leading cause of cancer-related mortalities (Forner et al., 2018). It has been reported that the highest incidence rates of HCC cases occur in Asia and Africa (Petrick et al., 2020), where the exposure to chronic hepatitis B is the main risk factor (Howell et al., 2021). Therapeutic options for the treatment of HCC have substantially evolved over the past 10 years. Nowadays, patients diagnosed with HCC at any stage of the disease can benefit from effective treatment, which greatly improves their survival rate. However, there are still several areas that need urgent improvement. The molecular mechanisms underlying tumorigenesis and the progression of HCC remain poorly understood (Zhao et al., 2019). At present, serum alpha-fetoprotein (AFP), ultrasonography, and CT scanning are still important means for the early diagnosis of HCC; however, the misdiagnosis rate is high (Kelley et al., 2020; Singal et al., 2020). Despite intensive research, the 5-year survival rate of HCC is still as low as <12% due to the lack of early detection strategy and effective therapy (Hlady et al., 2019). As a result, the investigation of effective prognostic biomarkers is a pivotal area among several considerations within the research of HCC.

DDOST encodes dolichyl-diphosphooligosaccharide–protein glycosyltransferase non-catalytic subunit that forms oligosaccharyltransferase (OST) complex, which catalyzes high-mannose oligosaccharides transferring to asparagine residues on nascent polypeptides in the lumen of the rough endoplasmic reticulum (ER) (Yamagata et al., 1997). A short cytosolic tail of DDOST has a functional ER-retention di-lysine motif, serving as a mechanism for retaining OST in the ER (Fu and Kreibich, 2000). DDOST also played a role in the processing of advanced glycation end products (AGEs), which are formed from non-enzymatic reactions between lipids or protein and sugars and are associated with aging and many diseases including the congenital disorders of glycosylation (Jones et al., 2012; Zhuang et al., 2017). AGEs and their receptor had been proven to be upregulated in liver fibrosis, and the silencing receptor of AGEs reduced collagen deposition and the tumor growth of HCC (Hollenbach, 2017). A previous study also revealed that the increased expression of DDOST was significantly associated with poorer clinical outcomes in cutaneous squamous cell carcinoma (Shapanis et al., 2021). Based on the those research, DDOST may play an important role in HCC. However, the prognostic potential of DDOST for HCC has not been reported.

## Materials and Methods

### Data Acquisition and Preprocessing

We utilized The Cancer Genome Atlas (TCGA) database (https://portal.gdc.cancer.gov/) for liver hepatocellular carcinoma (LIHC) to obtain the RNA-Seq data of 374 HCC patients accompanied with 50 normal tissues on gene expression, immune system infiltrates, and related patients’ clinical information (Blum et al., 2018). Then, we transferred RNAseq data in FPKM format to TPM format, retained clinical data and RNAseq data, and further analyzed all data in accordance with the publication guidelines provided by TCGA (https://www.cancer.gov/about-nci/organization/ccg/research/structural-genomics/tcga/using-tcga).

### Differentially Expressed Gene Analysis

The expression data (HTseq-Counts) were divided into high and low expression groups according to the median DDOST expression level and was then further analyzed by unpaired Student’s t-test within the DESeq2 R package (3.6.3) (Love et al., 2014). Adjusted *p* <0.05 and |log2-fold change (FC)| >1.5 were considered as thresholds for the DEGs.

### Enrichment Analysis

Gene ontology (GO) functional enrichment analysis and gene set enrichment analysis (GSEA) were all performed by ClusterProfiler package in R (3.6.3) (Yu et al., 2012). The DEGs between the high and low expression levels of DDOST were selected to be analyzed. GO analysis includes cellular component (CC), molecular function (MF), and biological process (BP). GSEA is a computational method to determine whether an *a priori* defined set of genes has statistical significance and concordant differences in two biological states. Additionally, the normalized enrichment score (NES) and adjusted *p*-value were utilized to sort the enriched pathways in each phenotype (Subramanian et al., 2005). C2. Cp.v7.2. symbols.gmt [Curated] was selected as the reference gene set of the KEGG pathway, C5. All.v7.2. symbols.gmt [Gene ontology] was selected as the reference gene set of GO term. Gene sets with a false discovery rate (FDR) <0.25 and adjusted *p* <0.05 were considered significantly enriched.

### Immune Infiltration Analysis

ssGSEA was realized by the GSVA package (Hänzelmann et al., 2013) in R to investigate the correlation between DDOST and the signature genes of 24 types of immune cells and then systematically analyzed the immune infiltrates of DDOST in the published literature (Bindea et al., 2013). The infiltration of immunocytes between the DDOST high and low expression group was analyzed by Spearman correlation and the Wilcoxon rank-sum test.

### Protein–Protein Interaction Network

The protein–protein interaction (PPI) network of co-regulated DEGs and the functional interaction between proteins were analyzed by the Search Tool for the Retrieval of Interacting Genes database (http://string-db.org) (Szklarczyk et al., 2019) and visualized by Cytoscape software (version 3.7.2). The combined score threshold of interaction in our study was 0.7. The database has a comprehensive score for each pair of protein relationships distributed between 0 and 1; the higher the total score, the more reliable the PPI relationship.

### Validation Analysis

The different DDOST expressions between HCC and non-tumor tissue was also analyzed in three RNAseq datasets (GSE87630, GSE101685, and GSE60502), which were downloaded from the GEO database (http://www.ncbi.nlm.nih.gov/geo).

The Kaplan–Meier (K-M) plotter is capable to assess the effect of 54 k genes on survival in 21 cancer types (http://kmplot.com/analysis/index.php?p=service&cancer=liver_rnaseq). The sources for the databases include GEO, EGA, and TCGA. The primary purpose of the tool is a meta-analysis-based discovery and validation of survival biomarkers (Menyhárt et al., 2018). DDOST was inputted in the K-M plotter to analyze the relationship between the expression of DDOST and the survival days of HCC patients, which were visualized in K-M survival plots. The log rank *p*-value <0.05 was considered statistically significant.

### Statistical Analysis

The statistical data acquired from TCGA were processed by R 3.6.3. The expression levels of DDOST between HCC and the normal group were compared by Wilcoxon rank-sum test and Wilcoxon signed-rank test. The correlation between DDOST expression and the grade of clinicopathological factors was analyzed by Welch one-way ANOVA, followed by the Bonferroni correction or *t*-test. The effect of the clinicopathological factors on DDOST expression was analyzed by univariate logistic regression, the Fisher exact test, and normal and adjusted Pearson κ^2^ tests. Moreover, we combined univariate Cox regression analysis and multivariate Cox regression analysis to evaluate the prognostic value of DDOST expression and other clinicopathological factors on overall survival (OS). All variables in the univariate analysis were put into the multivariate analysis. The K-M curve was drawn to evaluate the prognostic value of DDOST. The hazard risk (HR) of the individual for OS and disease-specific survival (DSS) were estimated by univariate Cox proportional hazard regressions. The HR of individual factors was estimated by measuring the HR with a 95% confidence interval (CI).

The receiver operating characteristic (ROC) analysis of DDOST was realized by the pROC package (Robin et al., 2011). The calculated area under the curve (AUC) value ranges, which were from 0.5 to 1.0, indicated the discrimination ability of 50%–100%. The time-dependent analysis of the ROC curve was constructed to evaluate DDOST for predicting the HCC outcome at 1, 3, and 5 years. All statistical tests were considered significant when two-tailed *p* ≤0.05.

## Result

### Clinical Characteristics

The clinical data of 374 HCC patients included the patients’ age, gender, T stage, N stage, M stage, pathologic stage, gender, age, histologic grade, vascular invasion, OS event, BMI, and AFP (ng/ml) (Table 1). A total of 253 males and 121 females were analyzed in the present study. The Fisher’s exact test result showed that DDOST was significantly correlated with OS event (*p* = 0.013); the chi-square test result revealed that DDOST had a trend of correlation with T stage (*p* = 0.074), histological grade (*p* = 0.077), and vascular invasion (*p* = 0.072). The Wilcoxon rank-sum test showed that DDOST was significantly correlated with AFP (ng/ml) (*p* < 0.001) and BMI (*p* = 0.031). DDOST expression was not significantly correlated with other clinicopathologic features.

**TABLE 1:** Demographic and clinicopathological parameters of high and low DDOST expression group patients with hepatocellular carcinoma in TCGA-LIHC.

Characteristic	Low expression of DDOST	High expression of DDOST	p
N	187	187	
T stage, n (%)			0.074
T1	104 (28%)	79 (21.3%)	
T2	40 (10.8%)	55 (14.8%)	
T3	37 (10%)	43 (11.6%)	
T4	5 (1.3%)	8 (2.2%)	
N stage, n (%)			0.622
N0	128 (49.6%)	126 (48.8%)	
N1	1 (0.4%)	3 (1.2%)	
M stage, n (%)			1.000
M0	133 (48.9%)	135 (49.6%)	
M1	2 (0.7%)	2 (0.7%)	
Pathologic stage, n (%)			0.129
Stage I	99 (28.3%)	74 (21.1%)	
Stage II	40 (11.4%)	47 (13.4%)	
Stage III	37 (10.6%)	48 (13.7%)	
Stage IV	3 (0.9%)	2 (0.6%)	
Gender, n (%)			0.269
Female	55 (14.7%)	66 (17.6%)	
Male	132 (35.3%)	121 (32.4%)	
OS event, n (%)			0.013
Alive	134 (35.8%)	110 (29.4%)	
Dead	53 (14.2%)	77 (20.6%)	
Vascular invasion, n (%)			0.072
No	118 (37.1%)	90 (28.3%)	
Yes	50 (15.7%)	60 (18.9%)	
Histologic grade, n (%)			0.077
G1	33 (8.9%)	22 (6%)	
G2	96 (26%)	82 (22.2%)	
G3	52 (14.1%)	72 (19.5%)	
G4	5 (1.4%)	7 (1.9%)	
Age, median (IQR)	62 (53, 69)	60.5 (51, 68)	0.306
AFP(ng/ml), median (IQR)	7.5 (3, 113.5)	27.5 (7, 738.75)	<0.001
BMI, median (IQR)	25.03 (22.42, 29.65)	24.16 (20.96, 28.03)	0.031

### Differential Expression Analysis of DDOST in HCC

**FIGURE 1:**
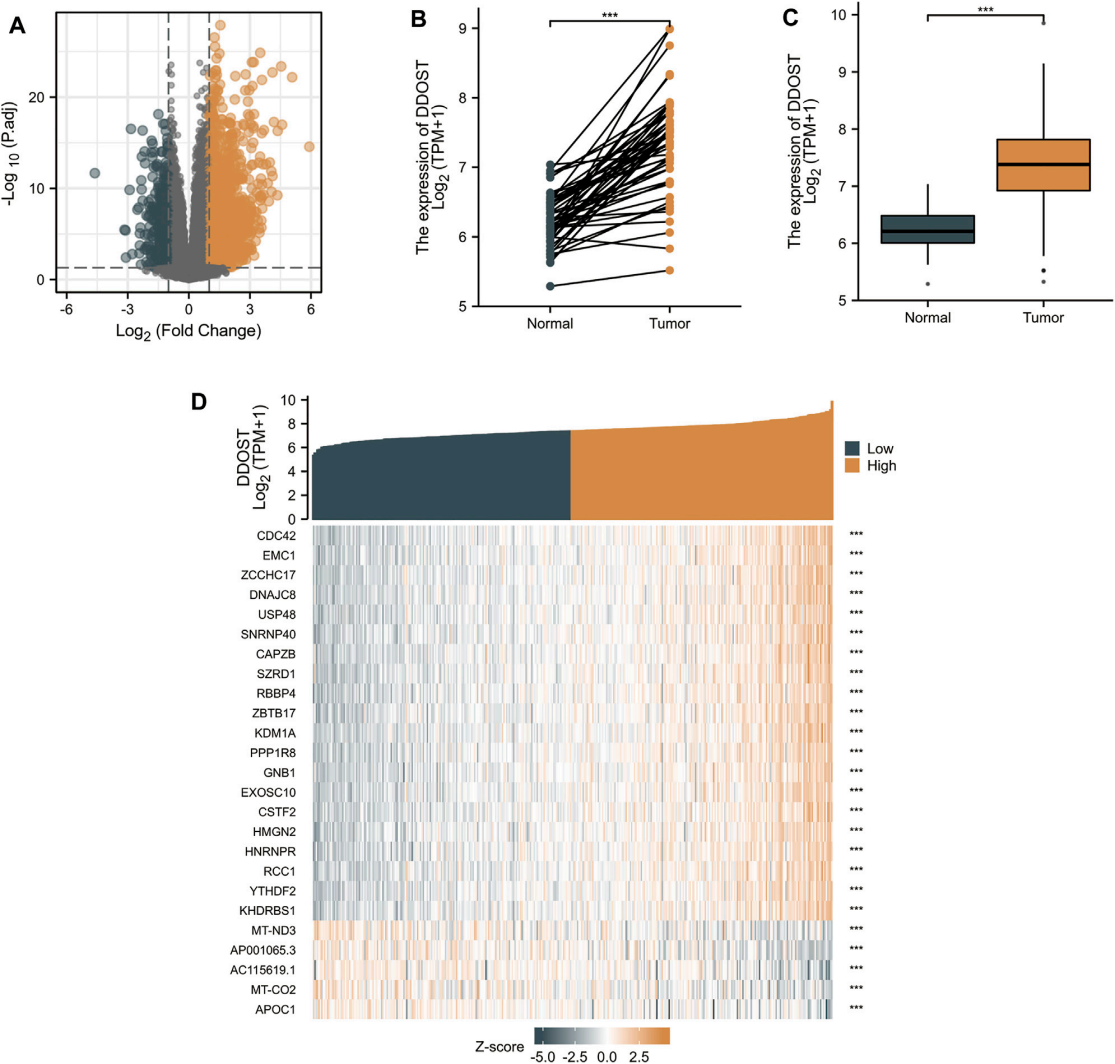
The results of differentially expressed gene (DEG) analysis. **(A)** The volcano plot of differentially expressed RNAs. **(B,C)** The different expressions of DDOST between HCC and the normal group. **(D)** The heat map of the 25 genes correlated to DDOST.

With |logFC| <1.5 and adjusted *p* < 0.05 set as the cut-off criteria, a total of 951 DEGs were identified (857 upregulated and 94 downregulated) by analyzing the HTSeq-Counts data of DDOST-related genes from TCGA. DEGs expressions were visualized in a volcano plot (Figure 1A). The unpaired and paired differential expression analyses between normal and HCC groups indicated that DDOST was expressed significantly higher in tumors compared to normal tissue (Figures 1B,C). The correlation between DDOST and 25 genes was demonstrated in a heat map (Figure 1D).

### Functional Enrichment Analysis of DEGs

**FIGURE 2:**
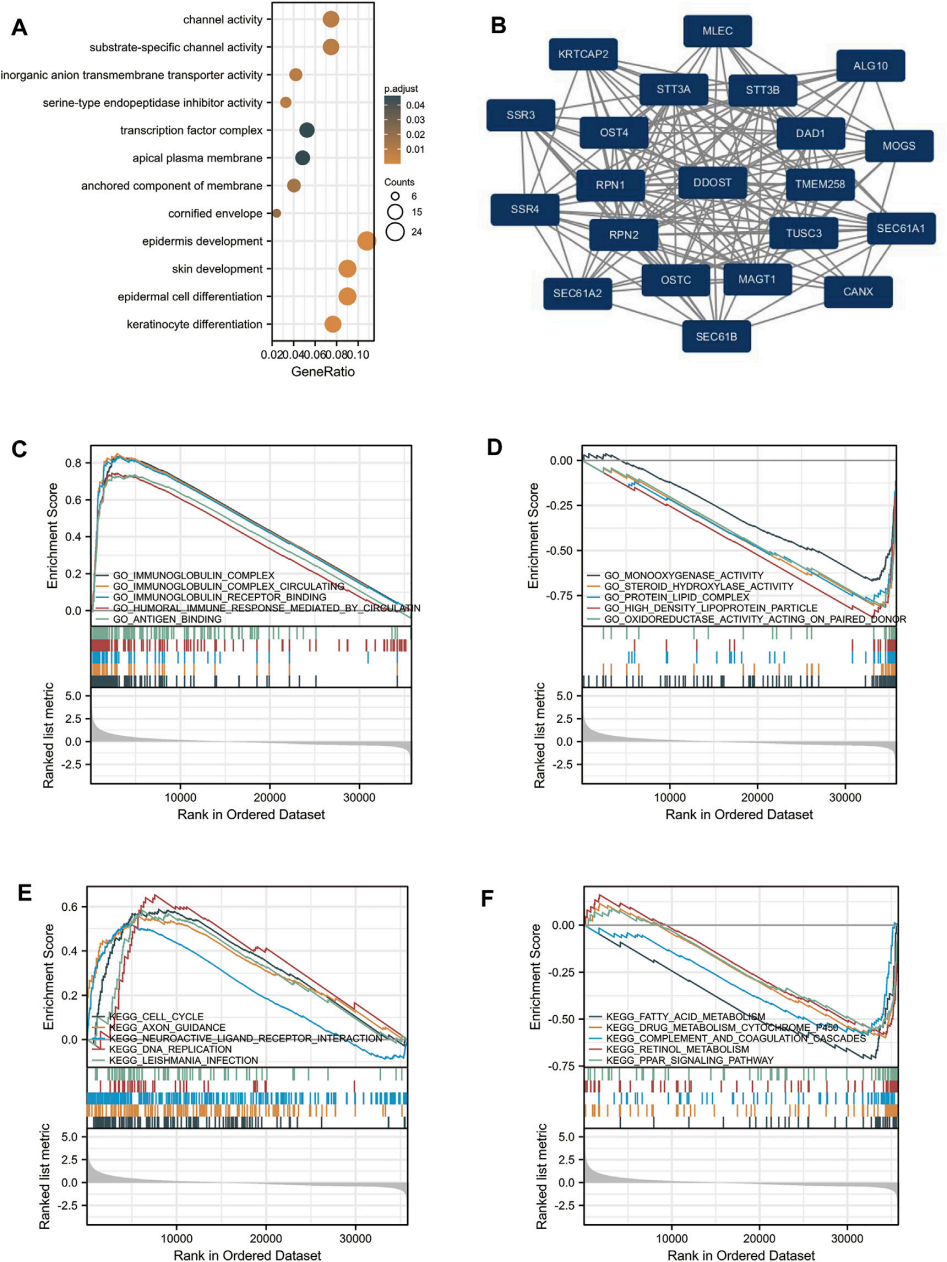
Enrichment analysis of DDOST in HCC. **(A)** Biological process enrichment related to DDOST-related genes. **(B)** A network of DDOST and its 20 potential co-interaction proteins. **(C–F)** The results of enrichment analysis from GSEA.

GO analysis indicated that DEG-related DDOST had significant regulation on epidermis development, skin development, epidermal cell differentiation, keratinocyte differentiation, channel activity, substrate-specific channel activity, inorganic anion transmembrane transporter activity, serine-type endopeptidase inhibitor activity, the anchored component of membrane, and cornified envelope (Figure 2A). The network of DDOST and its potential co-expression genes in DDOST-related DEGs are shown in Figure 2B.

To further identify the biological function of DDOST, the GSEA of differences between low and high DDOST expression data sets were performed to identify the GO term and KEGG pathway associated with DDOST. A total of 476 pathways showed significant differences (FDR <0.05, adjusted *p* < 0.05) in the enrichment of GO terms and KEGG pathways in samples with a high expression of DDOST. The most significantly enriched GO term and KEGG pathway based on their NES are shown in Table 2. The GSEA analysis in GO term revealed that immunoglobulin complex, immunoglobulin complex circulating, immunoglobulin complex receptor binding, humoral immune response mediated by circulating immunoglobulin, and antigen-binding were positively correlated with high levels of DDOST (Figure 2C); monooxygenase activity, steroid hydroxylase activity, protein–lipid complex, high-density lipoprotein particle, and oxidoreductase activity on paired donors were negatively correlated with high levels of DDOST (Figure 2D). The GSEA analysis in the KEGG pathway revealed that cell cycle, axon guidance, neuroactive ligand–receptor interaction, DNA replication, and *Leishmania* infection were positively correlated with high levels of DDOST (Figure 2E); Fatty acid metabolism, drug metabolism cytochrome P450, complement and coagulation cascades, retinol metabolism, and the PPAR signaling pathway were negatively correlated with high levels of DDOST (Figure 2F). These results indicate that the pathways regulating immunoglobulin complex, cell cycle control, and DNA replication were strongly associated with DDOST expression.

**TABLE 2:** Signaling pathways most significantly associated with DDOST expression.

	Description	NES	P-value	p.adjust
Positive GO term	GO_IMMUNOGLOBULIN_COMPLEX	3.258	0.001	0.020
	GO_IMMUNOGLOBULIN_COMPLEX_CIRCULATING	2.969	0.001	0.020
	GO_IMMUNOGLOBULIN_RECEPTOR_BINDING	2.933	0.001	0.020
	GO_HUMORAL_IMMUNE_RESPONSE_MEDIATED_BY_CIRCULATING_IMMUNOGLOBULIN	2.889	0.001	0.020
	GO_ANTIGEN_BINDING	2.886	0.001	0.020
Negative GO term	GO_MONOOXYGENASE_ACTIVITY	−3.133	0.006	0.037
	GO_STEROID_HYDROXYLASE_ACTIVITY	−3.069	0.003	0.027
	GO_PROTEIN_LIPID_COMPLEX	−3.013	0.003	0.027
	GO_HIGH_DENSITY_LIPOPROTEIN_PARTICLE	−3.002	0.003	0.027
	GO_OXIDOREDUCTASE_ACTIVITY_ACTING_ON_PAIRED_DONORS	−2.988	0.003	0.027
Positive KEGG term	KEGG_CELL_CYCLE	2.273	0.001	0.025
	KEGG_AXON_GUIDANCE	2.173	0.001	0.025
	KEGG_NEUROACTIVE_LIGAND_RECEPTOR_INTERACTION	2.147	0.001	0.025
	KEGG_DNA_REPLICATION	2.084	0.003	0.026
	KEGG_LEISHMANIA_INFECTION	2.080	0.001	0.025
Negative KEGG term	KEGG_FATTY_ACID_METABOLISM	−2.817	0.004	0.027
	KEGG_DRUG_METABOLISM_CYTOCHROME_P450	−2.614	0.005	0.029
	KEGG_COMPLEMENT_AND_COAGULATION_CASCADES	−2.571	0.005	0.029
	KEGG_RETINOL_METABOLISM	−2.544	0.005	0.029
	KEGG_PPAR_SIGNALING_PATHWAY	−2.441	0.005	0.029

### Relationship Between DDOST Expression and Immune Infiltration

**FIGURE 3:**
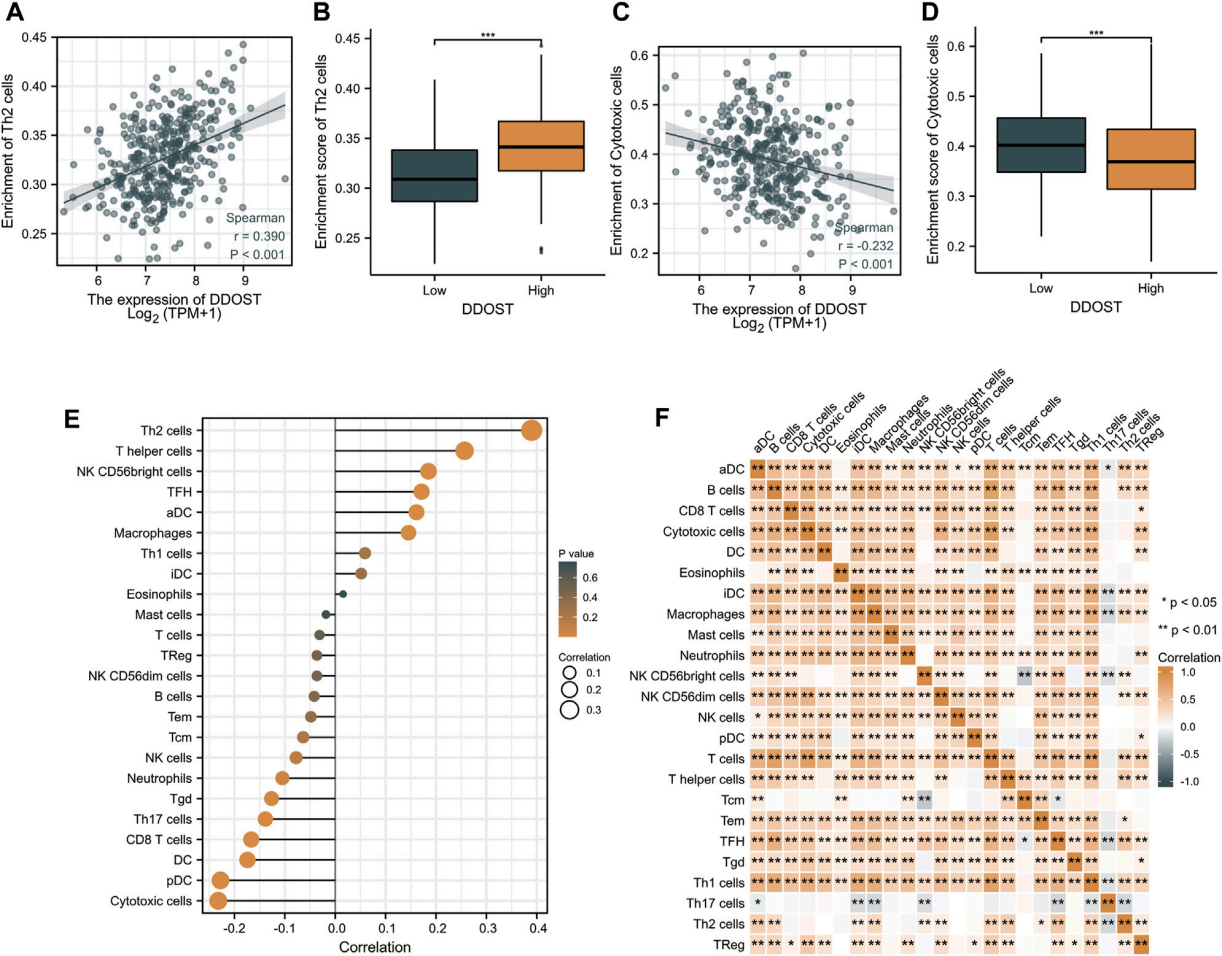
The results of analysis between DDOST expression and immune infiltration. **(A)** The positive correlation between DDOST expression and Th2 cells. **(B)** Th2 cells’ infiltration level in different DDOST expression groups. **(C)** The negative correlation between DDOST expression and cytotoxic cells. **(D)** Cytotoxic cells’ infiltration level in different DDOST expression groups. **(E)** Correlation between DDOST expression level and the relative abundances of 24 immune cells. **(F)** Heat map of 24 immune infiltration cells in HCC.

Spearman correlation was employed to study the correlation between the DDOST expression level in TPM format and the immune cell infiltration level quantified as the ssGSEA score. The Th2 cells’ infiltration level displays a significantly positive correlation with DDOST expression (Spearman R = 0.390, *p* < 0.001) (Figure 3A) and was significantly higher in the DDOST high-expression group (*p* < 0.001) (Figure 3B). On the other hand, the cytotoxic cells’ infiltration level showed a significantly negative correlation with DDOST expression (Spearman R = -0.232, *p* < 0.001) (Figure 3C) and was significantly lower in the DDOST high-expression group (*p* < 0.001) (Figure 3D). T helper cells, NK CD56bright cells, TFH, aDC, and macrophages have also shown a positive relation with DDOST. pDC, DC, CD8 T cells, Th17 cells, Tgd, neutrophils, and NK cells have shown a negative correlation with DDOST (Figure 3E). These results indicated the vital role of DDOST in the immune infiltration in HCC. Different degrees of correlation between the ratios of 24 types of different tumor-infiltrating immune cells’ subpopulations were assessed and visualized by a heat map (Figure 3F).

### Associations Between DDOST Expression and Clinicopathologic Variables

**FIGURE 4:**
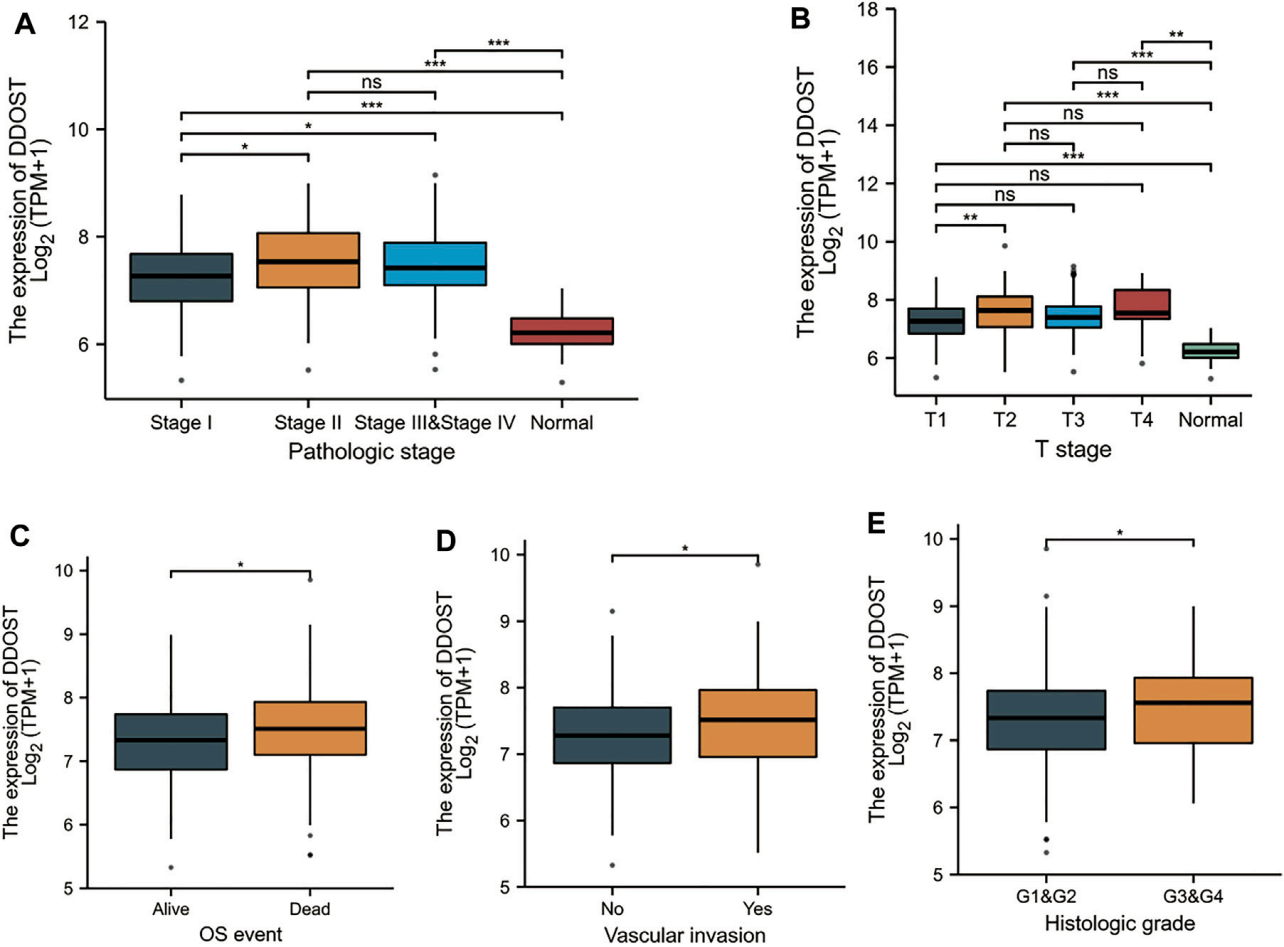
Association between the DDOST expression and different clinicopathologic characteristics. **(A)** Association between the DDOST expression and the pathologic stage of HCC, **(B)** T stage, **(C)** OS event, **(D)** vascular invasion, and **(E)** histologic grade.

Welch one-way ANOVA followed by the Bonferroni correction proved that the expression of DDOST was significantly correlated with the pathologic stage and T stage (Figures 4A,B). The *t*-test revealed that the expression of DDOST was significantly correlated with the histologic grade, vascular invasion, and OS event (Figures 4C–E). Logistic regression analysis showed that DDOST was significantly correlated with the T stage (*p* = 0.011) and histologic grade (*p* < 0.013) and had a trend of correlation with vascular invasion (*p* = 0.056) (Table 3).

**TABLE 3:** DDOST expression correlated with clinicopathological characteristics analyzed by logistic regression.

Characteristics	Total (N)	Odds ratio (OR)	*p*-Value
T stage (T2 and T3 and T4 vs. T1)	371	1.702 (1.130–2.572)	0.011
N stage (N1 vs. N0)	258	3.048 (0.384–62.060)	0.337
M stage (M1 vs. M0)	272	0.985 (0.117–8.308)	0.988
Pathologic stage (Stage III and Stage IV vs. Stage I and Stage II)	350	1.436 (0.888–2.333)	0.141
Histologic grade (G3 and G4 vs. G1 and G2)	369	1.719 (1.123–2.644)	0.013
Vascular invasion (Yes vs. No)	318	1.573 (0.990–2.511)	0.056
AFP (ng/ml) (>400 vs. ≤400)	280	1.598 (0.916–2.808)	0.100
Albumin (g/dl) (≥3.5 vs. <3.5)	300	0.751 (0.437–1.287)	0.297
Tumor status (with tumor vs. tumor-free)	355	1.301 (0.854–1.985)	0.221

**FIGURE 5:**
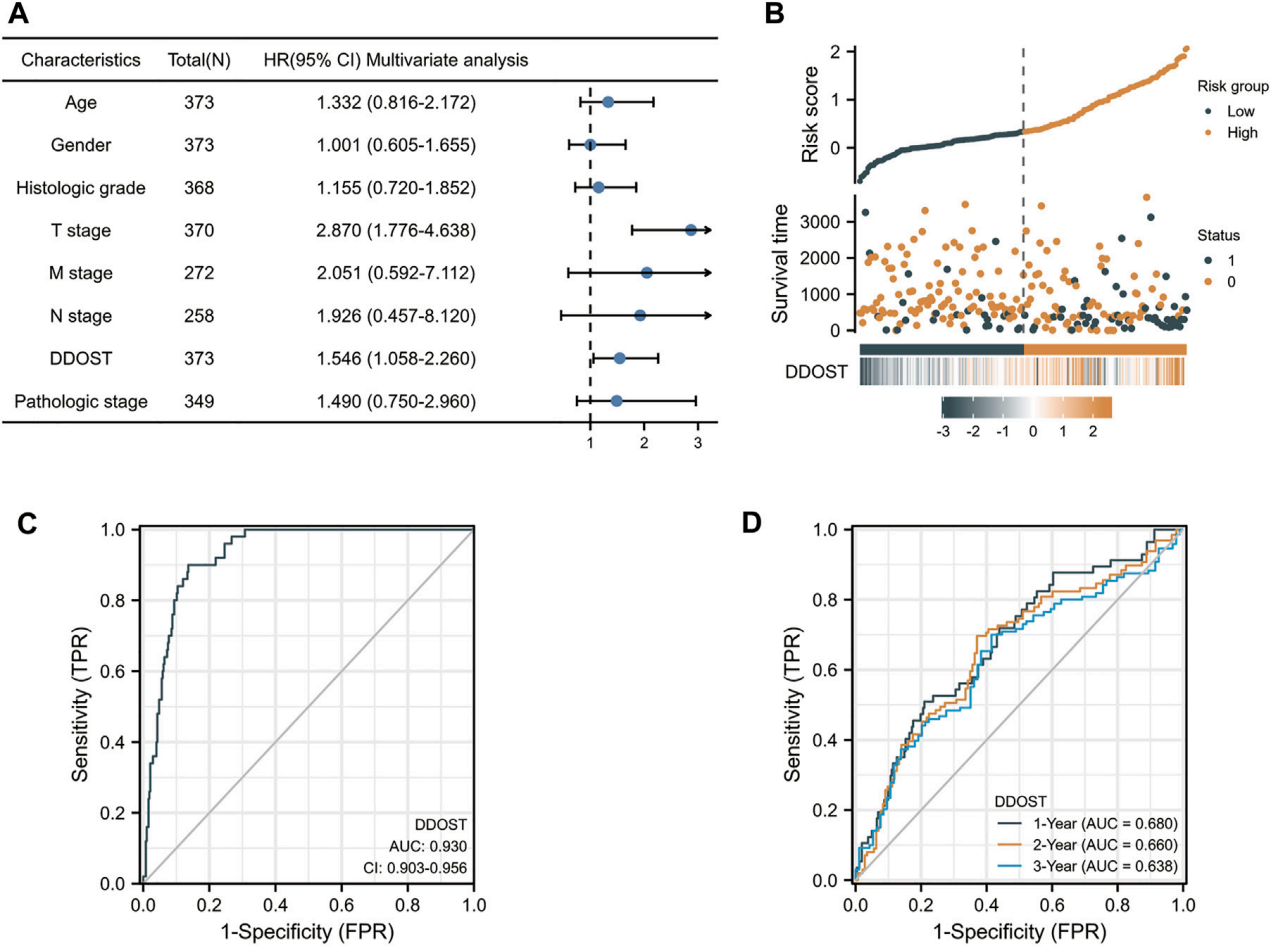
The prognostic value of DDOST in LIHC. **(A)** Multivariate Cox regression visualized in the forest plot **(B)** DDOST expression distribution and survival status. 0: dead, 1: alive. **(C)** Diagnostic ROC curve of DDOST. **(D)** Time-dependent ROC curve of DDOST.

In the Cox regression model, univariate Cox regression indicates that the T stage (*p* < 0.001), M stage (*p* = 0.017), pathologic stage (*p* < 0.001), and DDOST (*p* < 0.001) were correlated with the bad prognosis of HCC (Table 4). All variables in univariate Cox regression were included in multivariate Cox regression. Multivariate Cox regression showed that T stage (*p* = 0.017) and DDOST (*p* = 0.038) were independent prognostic factors for OS (Figure 5A).

**TABLE 4:** Univariate and multivariate analyses of clinical pathological parameters in HCC patients.

Characteristics	Total (N)	Univariate analysis		Multivariate analysis	
		Hazard ratio (95% CI)	*p*-Value	Hazard ratio (95% CI)	*p*-Value
Age	373				
≤60	177	Reference			
>60	196	1.205 (0.850–1.708)	0.295	1.323 (0.811–2.159)	0.262
Gender	373				
Female	121	Reference			
Male	252	0.793 (0.557–1.130)	0.200	0.993 (0.597–1.652)	0.979
Histologic grade	368				
G1	55	Reference			
G2	178	1.162 (0.686–1.968)	0.577	0.810 (0.390–1.685)	0.573
G3 and G4	135	1.222 (0.710–2.103)	0.469	1.003 (0.489–2.058)	0.993
T stage	370				
T1 and T2	277	Reference			
T3 and T4	93	2.598 (1.826–3.697)	**<0.001**	2.183 (1.150–4.141)	**0.017**
M stage	272				
M0	268	Reference			
M1	4	4.077 (1.281–12.973)	**0.017**	2.152 (0.615–7.535)	0.231
N stage	258				
N0	254	Reference			
N1	4	2.029 (0.497–8.281)	0.324	1.561 (0.357–6.826)	0.554
DDOST	373	1.585 (1.241–2.026)	**<0.001**	1.491 (1.022–2.176)	**0.038**
Pathologic stage	349				
Stage I	173	Reference			
Stage II and Stage III and Stage IV	176	2.090 (1.429–3.055)	**<0.001**	1.490 (0.750–2.960)	0.255

Bold values were statistically significant

The distribution of DDOST expression, survival status of HCC patients, and expression profiles of DDOST are shown in Figure 5B. The blue dots represent the surviving HCC patients, and the orange dots represent the dead HCC patients. The upper line represents the median of risk score. The left side of the upper line represents the low-risk score group with a low expression of DDOST, and the right side of the dotted line represents the high-risk score group with a high expression of DDOST. With the increase of risk score in HCC patients, the number of orange dots increased gradually, and the number of dead HCC patients increased. It shows that the patients in the high-risk group have poorer survival and a higher risk of death.

The ROC analysis of DDOST supported the diagnostic accuracy of the score (AUC = 0.93, 95% CI: 0.903–0.956) (Figure 5C). The time-dependent accuracy of DDOST in predicting OS in 1, 2, and 3 years was also assessed through a time-dependent ROC analysis (Figure 5D).

**FIGURE 6:**
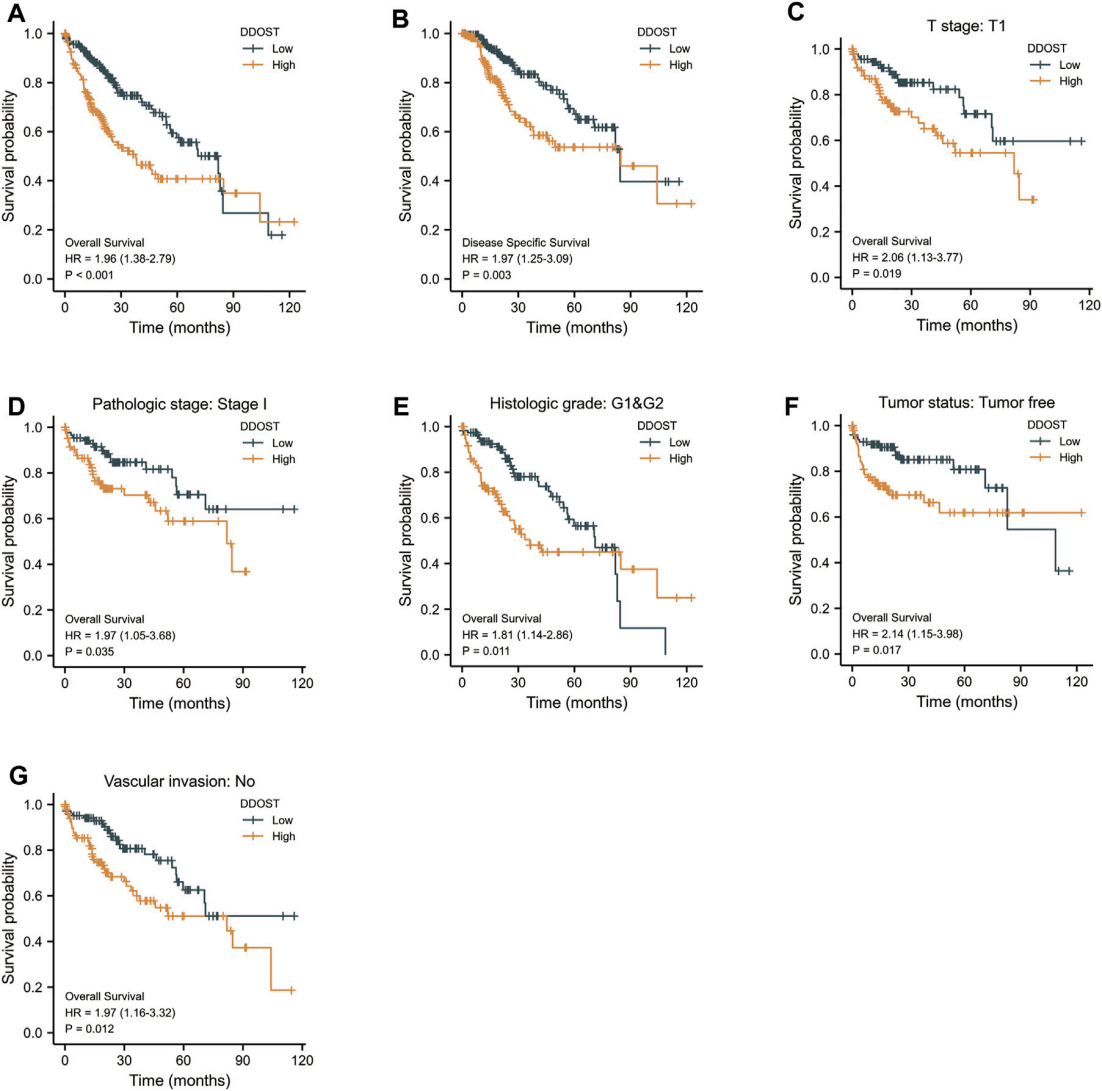
The prognostic value of DDOST in the different subgroups. **(A,B)** The prognostic value of DDOST in OS and DSS of HCC. **(C–G)** High expression of DDOST was associated with worse OS in different subgroups.

The K-M survival curve drawn by survminer package in R was used to evaluate the prognostic value of DDOST in OS of HCC. HCC patients were divided into high and low expression groups based on the DDOST expression median value. The high expression group has a strong correlation with worse OS (HR = 1.96 (1.38–2.79), *p* < 0.001) and DSS (HR = 1.97 (1.25–3.09), *p* = 0.003) (Figures 6A,B).

The high expression of DDOST was also associated with worse OS in the T1 subgroup of T stage (HR = 2.06 (1.13–3.77), *p* = 0.019), stage I subgroup of pathologic stage (HR = 1.97 (1.05–3.68), *p* = 0.035), G1 and G2 subgroup of histologic grade [HR = 1.81 (1.14–2.86), *p* = 0.011], tumor- free subgroup of tumor status (HR = 2.14 (1.15–3.98), *p* = 0.017), and no vascular invasion subgroup of vascular invasion (HR = 1.97 (1.16–3.32), *p* = 0.012) (Figures 6C–F).

### Data Validation

In all three GEO datasets, DDOST mRNA expression exhibited a significant increase in HCC when compared to the normal group (*p*-value < 0.01, Supplement Figures 1A–C). K-M survival plots also showed the group with high DDOST expression having poor OS rates (log rank *p*-value = 0.010, Supplement Figure 1D).

## Discussion

As far as we know, the majority of the membrane and secretory proteins synthesized in the ER are modified with N-glycans in eukaryotes. The N-glycosylation reaction catalyzed by OST had been implicated in cell-to-cell communication, signal transduction, trafficking, folding, and the degradation of proteins (Ohtsubo and Marth, 2006; Harada et al., 2015; Mikolajczyk et al., 2020) and was involved in the mechanism of tumor immune escape in the tumor microenvironment (Hsu et al., 2018). Thus, OST can be a potential therapeutic target for cancer treatment (Harada et al., 2019). RPN2, TUSC, as well as DDOST are the subunits of OST. The expression of RPN2 is positively correlated with the progression of breast cancers (Ono et al., 2015), non-small cell lung (Fujita et al., 2015), gastric (Fujimoto et al., 2018), esophageal (Li et al., 2019), and colorectal cancers (Bi and Jiang, 2018), whereas TUSC3 was reported as a candidate tumor suppressor (Vašíčková et al., 2018). DDOST, which also acted as advanced glycation end product-receptor 1 (Li et al., 1996), had been reported in regulating AGE, which increased oxidative stress and inflammation and may be involved in liver injury and subsequent carcinogenesis (Moy et al., 2013). In this research, we analyzed the sequencing data on liver cancer patients from TCGA to study the potential function and evaluate the prognostic value of DDOST.

DDOST is highly expressed in HCC patients and correlated with several advanced clinical features (pathological stage, T stage histologic grade, vascular invasion, OS event), which suggested that DDOST is a potential prognostic and diagnostic marker deserving further clinical validation. The function of DDOST in HCC was further investigated in GSEA using TCGA data.

The PPI network indicated that DDOST can interact with SSR3, SSR4, SEC61A1, SEC61A2, SEC61B, CANX, MOGS, ALG10, and MLEC other than the subunit protein of OST. All those proteins were closely associated with the N-linked oligosaccharide processing pathway, which had long been considered directly associated with the metastatic potential of malignant tumor cells (Dennis, 1991). GSEA showed that positively enriched GO terms including immunoglobulin complex, immunoglobulin complex circulating, immunoglobulin complex receptor binding, humoral immune response mediated by circulating immunoglobulin, and antigen-binding were pivotal in immune complex formation. It is indicated that DDOST might participate in the immune response in the tumorigenesis of HCCs. On the other hand, the KEGG pathway analysis indicated that cancer-related pathways including cell cycle and DNA replication were positively enriched when DDOST was highly expressed. Furthermore, GSEA analysis revealed the vital role of DDOST in the metabolism of protein and fatty acids, and in the downregulation of the PPAR signaling pathway, which plays a vital role in protecting the liver from oxidation, inflammation, fibrosis, and tumors (Wu et al., 2020). Based on the results, we can presume that as a crucial molecular in regulating protein and lipid metabolism, the high DDOST expression may induce an immune response and regulate cell cycles in HCC by suppressing the PPAR pathway.

ssGSEA combined with Spearman correlation was adopted to investigate the relationship between DDOST expression and immune infiltration levels in HCC. Our results demonstrate that DDOST expression has a significantly positive correlation with Th2 cells and a strong-to-moderate correlation with T helper cells, NK CD56 bright cells, Tfh, aDC, macrophages, and Th1 cells. Our results indicate that a shift of Th1/Th2 balance toward Th2, which plays a vital role in HCC metastasis (Budhu and Wang, 2006), may be caused by the DDOST high expression. Th2 cell is one type of T helper cell that can induce the polarization of M1 macrophages into immunosuppressive M2 macrophages (DeNardo et al., 2009), and lead to the inhibition of the host immune system, hence contributing to tumorigenesis. IL-4 produced by Th2 cells can result in the activation of several cancer-related pathways (Zhao et al., 2015; Dey et al., 2020). Tfh cells can differentiate into Th1 and Th2 cells and regulate humoral immune response (Rezende et al., 2018). Based on previous research, we can conclude that overexpression of DDOST may induce immune infiltration in HCC genesis and progression.

There is an inverse correlation between cytotoxic cells, pDC, DC, CD8 T cells, Th17 cells, Tgd, neutrophils, NK cells, and DDOST. Cytotoxic cells including NK cells play a vital role in anti-tumor immunity. NK cells are important in innate immune surveillance against cancer (Lanier, 2005). CD8^+^ T cells exhibit a cytotoxic ability against tumor cells through differentiating cytotoxic T cells (Iwahori, 2020). DCs including pDC were essential contributors to immune defenses against cancer. IFN-I produced by pDC shows good anti-tumor activity (Saulep-Easton et al., 2014). Th17 cells were closely related to neutrophils (Amicarella et al., 2017), and they are critical in tumor immunity and predict a poor prognosis in HCC (Wang et al., 2020). The downregulation of those types of immune cells may facilitate the progression of HCC. All findings according to ssGSEA exhibited the important role of DDOST in regulating immune infiltration in HCC.

As a traditional serological marker, AFP has been adopted in the diagnosis of HCC for decades (Wong et al., 2014). However, AFP was not significant in all HCC cases. It is reported that only 60%–70% of total HCC patients have elevated AFP levels, and nonspecific increases are also observed in non-HCC diseases such as chronic hepatitis or liver cirrhosis (Akeyama et al., 1972; Di Bisceglie and Hoofnagle, 1989). More importantly, AFP levels are usually normal in early HCC (Chen et al., 1984). On the contrary, DDOST is highly expressed in early-stage HCC. Furthermore, compared with the DDOST low-level expression group, HCC patients with DDOST highly expressed the result in poor OS and DSS. The Cox HR model also suggested that DDOST was strongly associated with the OS in HCC. The relation between DDOST and those prognostic indicators suggested that DDOST was a powerful prognostic biomarker in HCC.

Overall, the important role of DDOST in HCC was revealed through our study. Our work demonstrated that increased expression of DDOST is associated with poor OS in HCC patients. GSEA showed that pathways including DNA replication, cell cycle, immune response in cancer, the PPAR signaling pathway, and lipid acid metabolism were associated with DDOST expression. Moreover, the connection between DDOST and tumor-infiltrating immune cells was identified. The work presented here provides a detailed analysis of the role of DDOST in HCC development, which will aid in the understanding of the mechanisms underlying HCC. Combined with a previous study, our research indicates that DDOST can affect protein and lipid metabolism by joining in nascent polypeptide processing in ER and regulating the AGE level; then, it may lead to PPAR pathway suppression and play an important role in cell cycle regulation and immune infiltration in HCC.

Although the vital role of DDOST in the regulation of the cell cycle and immune response in the tumorigenesis of HCC had been proven, *in vitro* and *in vivo* experiments are still needed to verify the correlation between DDOST expression and HCC development and then to illustrate the biological mechanism of DDOST in HCC progression. Clinical researches are required to evaluate the relationship between DDOST expression and clinical features including the HCC stage, and prognosis value, which might facilitate the identification of new markers for assessing the tumor progression, promoting drug development, and improving treatment strategy.

## Data Availability

The datasets presented in this study can be found in online repositories. The names of the repository/repositories and accession number(s) can be found in the article/Supplementary Material.
