# Supplementary figures and images for: Prognostic Biomarker DDOST and Its Correlation With Immune Infiltrates in Hepatocellular Carcinoma
Source: Front Genet. 2022 Jan 31;12:819520. doi: 10.3389/fgene.2021.819520 (PMC8841838; doi:10.3389/fgene.2021.819520)

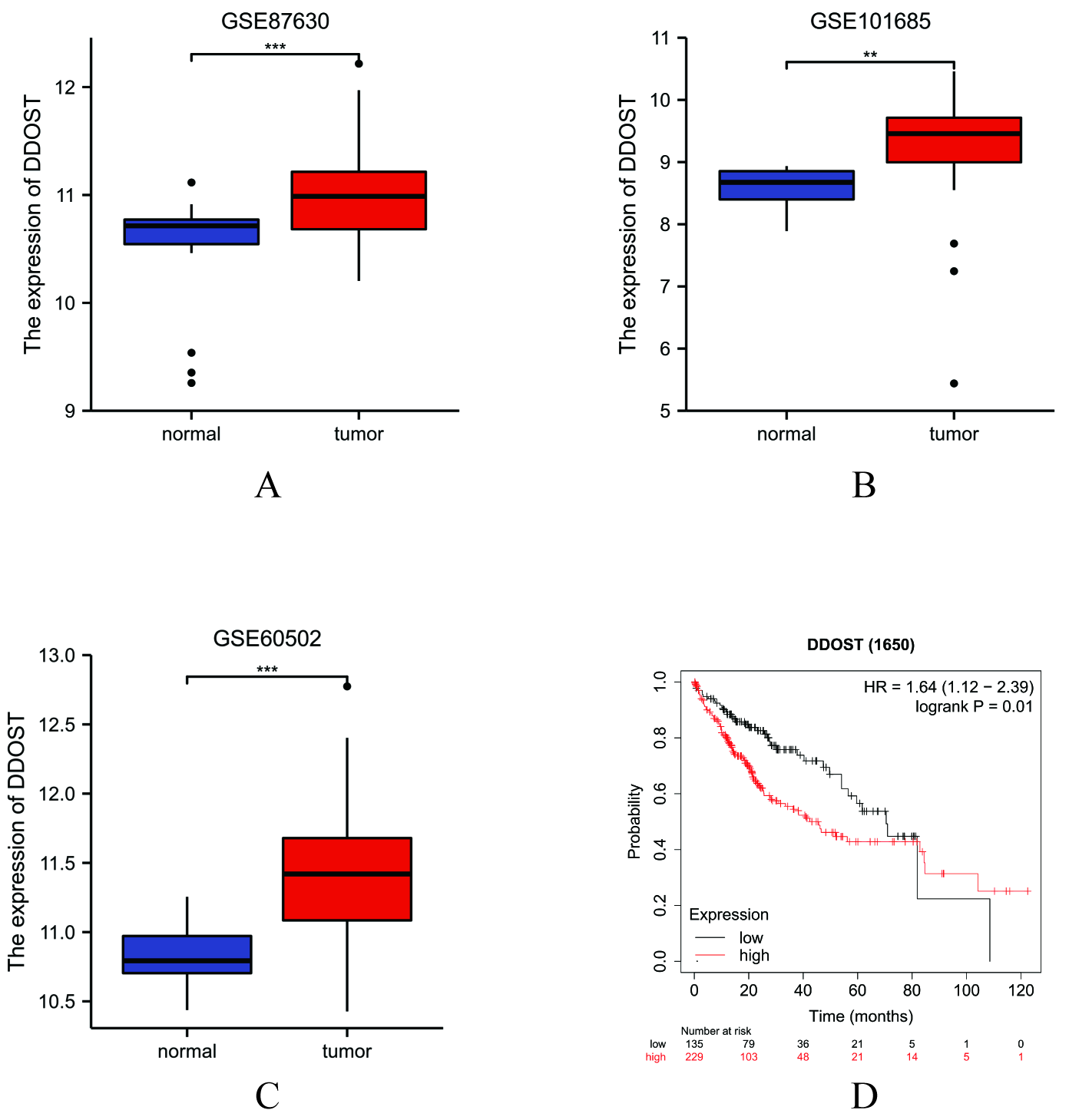

Supplement: Supplementary file 1 [file Image1.TIF]
